# Integrative multidisciplinary oncological approach in cancer patients receiving oncological treatments: a case series

**DOI:** 10.3389/fphar.2026.1741148

**Published:** 2026-05-14

**Authors:** Giuliana Ciappina, Giordana Di Mauro, Mariapia Marafioti, Fabiola De Luca, Gaetano Facchini, Benedetta Raneri, Emanuela Esposito, Mariacarmela Santarpia, Liliana Montella, Massimiliano Berretta

**Affiliations:** 1 Department of Medical Sciences, Section of Experimental Medicine, University of Ferrara, Ferrara, Italy; 2 Department of Chemical, Biological, Pharmaceutical and Environmental Sciences, PhD School in Applied Biology and Experimental Medicine, University of Messina, Messina, Italy; 3 Department of Human Pathology “G. Barresi”, School of Specialization in Medical Oncology, University of Messina, Messina, Italy; 4 Department of Biomedical, Dental and Morphological and Functional Imaging Sciences, PhD School in Translational Molecular Medicine and Surgery, University of Messina, Messina, Italy; 5 Department of Chemical, Biological, Pharmaceutical and Environmental Sciences, University of Messina, Messina, Italy; 6 Division of Medical Oncology, “Santa Maria delle Grazie” Hospital, Pozzuoli, Italy; 7 Medical Oncology Unit, Department of Human Pathology “G. Barresi”, University of Messina, Messina, Italy; 8 Department of Clinical and Experimental Medicine, University of Messina, Messina, Italy; 9 Division of Medical Oncology, “G. Martino” University Hospital, University of Messina, Messina, Italy; 10 Integrative Medicine Research Group (IMRG), Noceto, Italy

**Keywords:** cancer, chemotherapy side effects, complementary and alternative medicine, integrative medicine, mycotherapy, neutropenia

## Abstract

**Background:**

Complementary and integrative medicine (CIM) practices are increasingly utilized by cancer patients to alleviate treatment-related side effects and support immune function. Among these, mycotherapy has gained attention for its potential hematologic and immunomodulatory benefits.

**Methods:**

We report a retrospective observational case series of six cancer patients receiving standard oncologic therapy who were supplemented with a commercially available herbal preparation as part of routine integrative supportive care. Neutrophil count and patient-reported quality of life were monitored during standard clinical follow-up.

**Results:**

During supplementation, a temporal association was observed between integrative treatment and stabilization of neutrophil count, allowing continuation of anticancer therapy without further dose reductions or prolonged interruptions. No adverse events attributable to the supplement were recorded.

**Discussion:**

Although limited by its small sample size and observational design, this case series suggests that integrative mycotherapy may represent a feasible supportive approach in selected patients experiencing treatment-related hematologic toxicity.

**Conclusion:**

In this case series study, neutrophil recovery was observed with a temporal association between the herbal preparation and the outcome. These findings should be interpreted as hypothesis generating. Prospective controlled studies are warranted to clarify efficacy, safety, and optimal integration strategies within standard oncology care.

## 1 Introduction

In recent years, increasing attention has been paid to the field of complementary and integrative medicine (CIM), an area that is becoming increasingly important in both scientific debate and clinical practice. Complementary medicine encompasses a heterogeneous group of health-related interventions such as dietary supplements, vitamins, minerals, probiotics, and other natural products, acupuncture, phytotherapy, meditation, and yoga, that fall outside the scope of conventional biomedical practice but are often used in conjunction with it. When supported by solid clinical evidence, these practices can help to strengthen conventional medical approaches and address patients’ needs in a more comprehensive and holistic way (Mortada, 2024). In this framework, integrative medicine is defined as a multidisciplinary and evidence-based model of care that combines biomedical knowledge with validated practices from traditional and complementary medicine. Its main goal is to provide patient-centered care that simultaneously takes into account the biological, psychological and social dimensions of health and wellbeing (World Health Organization, 2013). Several institutions and academic centers around the world are currently investigating the clinical benefits of integrative medicine in various areas, including pain management, fatigue reduction and symptom relief in oncology patients and survivors. Despite remarkable advances in oncology therapies in recent decades, including chemotherapy (CT), immunotherapy, targeted agents, hormone-based treatments, antibody-drug conjugates (ADCs) and radiation therapy (RT), cancer treatment remains associated with a wide range of treatment-related toxicities. The most common adverse events include myelosuppression, gastrointestinal toxicity, alopecia, chronic fatigue, infectious complications, cardiotoxicity and neurotoxicity, which can significantly impact both adherence to prescribed treatments and overall quality of life (QoL) (Zafar et al., 2025). This scenario helps to explain the high prevalence of CIM use in cancer patients. As highlighted in previous studies, patients are more likely to resort to complementary practices for two main reasons: (i) dissatisfaction with treatment outcomes or prognosis, which prompts them to explore additional options, and (ii) the burden of treatment-related side effects, which often leads them to seek supportive measures that are believed to alleviate symptoms and improve functional status (Berretta et al., 2017). However, the true prevalence of CIM use in oncology remains difficult to ascertain, as patients often conceal such practices from their oncologists. This underreporting contributes to the likely underestimation of CIM use rates. Nevertheless, available data suggest that approximately 50% of breast cancer patients use some form of CIM during the course of their disease (Stöcker et al., 2023). The increasing use of CIM in oncology has led to a growing number of randomized controlled trials (RCTs) investigating its safety, efficacy and clinical impact (Greenlee et al., 2017; Mao et al., 2022; Zhang et al., 2023; Lopez et al., 2024). Preliminary results suggest that CIM, when appropriately integrated into conventional oncology care, can improve symptom management, increase the tolerability of medical therapies and positively impact QoL parameters (Berretta et al., 2025). However, it is crucial that such practices are based on scientific evidence and integrated into treatment under the supervision of healthcare professionals to ensure safety and avoid potential pharmacological interactions (Berretta et al., 2025). We present here the case of six patients with cancer who were receiving active oncologic treatment. These patients had persistent hematologic toxicities especially neutropenia and were supported by experienced physicians with CIM instead of discontinuing oncologic therapy. The integration of mycotherapy was associated with a favorable course of neutrophil counts in the six patients included in this case series. The use of mycotherapy alongside oncologic treatments showed a potential role in supporting neutrophil recovery and may represent a complementary strategy that could help patients maintain adherence to cancer treatments. However, these observations should be interpreted cautiously given the descriptive nature of the study.

## 2 Methods

### 2.1 Patients

As of 2021 a group of oncologist’s experts in CIM provided multi-target supportive treatments for 54 patients who required them. The written informed consent was already collected in a previous retrospective study (Berretta et al., 2025). All potentially identifying information has been omitted. Data on age, disease, stage and molecular profile, treatment regimen and setting, drug dose reduction, adverse drug reactions and response were retrieved from the electronic medical record after patients were reported by the prescribing oncologist. Toxicities were recorded retrospectively and categorized according to the Common Terminology Criteria for Adverse Events (CTCAE) Version 5.0. The patients were thoroughly informed about the proposed treatment, including all relevant details regarding the potential benefits, risks, and alternatives. The process of data collection and analysis for these cases was carried out following the ethical guidelines outlined in the Declaration of Helsinki, ensuring that the study adhered to internationally recognized standards for research involving human participants.

### 2.2 Mycotherapy U-CARE

Mycotherapy U-CARE (owned by AVD Reform srl, Noceto, Parma, Italy) is a medicinal mushroom extract mixture and has been registered by the Italian Ministry of Health as a dietary supplement (registration number 627 I.5.i.h.2/2020/627). It is produced from concentrated hydroalcoholic extracts of five medicinal mushrooms, obtained through hot water extraction, ethanol precipitation, and subsequent freeze-drying. The preparation contained the following fungal species (taxonomically validated according to Index Fungorum): Ganoderma lucidum (Curtis) P. Karst [Ganodermataceae]; Grifola frondosa (Dicks.) Gray [Meripilaceae]; Agaricus subrufescens Peck (syn. Agaricus blazei Murrill) [Agaricaceae]; Ophiocordyceps sinensis (Berk.) G.H. Sung et al. (syn. Cordyceps sinensis) [Ophiocordycipitaceae]; Lentinula edodes (Berk.) Pegler [Omphalotaceae] in equal amounts (150 mg each). Each capsule thus contains β-(1,3)-D-glucans and β-(1-6)-D-glucans (exceeding 15%), and polysaccharides greater than 30% and other bioactive compounds from the medicinal mushroom mixture (Table 1). Micotherapy U-Care is a commercially available formulation composed of standardized extracts derived from medicinal mushrooms. In particular, according to the manufacturer’s technical documentation, the formulation contains extracts obtained from the fruiting bodies of five medicinal mushrooms mentioned above. The fungal strain was obtained from the fungal culture collection of MycoMedica d.o.o. (Slovenia) and initially cultivated on Potato Dextrose Agar (PDA) under dark conditions at 24 °C. After approximately 20 days of growth, cultures were transferred onto lignocellulosic substrates and incubated for a further 60 days at the same temperature to allow the development of fruiting bodies. At the end of the incubation period, the fruiting bodies were harvested and subjected to hydroalcoholic extraction using water and ethanol as solvents, with a solvent-to-material ratio of 15:1 (w/v) for 3 h. The resulting extract was then dried under vacuum (70 °C, −0.9 bar) and finely milled using a UPZ mill (Hosokawa Alpine Aktiengesellschaft, Augsburg, Germany) to obtain particles predominantly smaller than 100 μm. The extract was obtained with a drug-to-extract ratio (DER) of 10:1, corresponding to approximately 1 kg of dry powdered extract derived from 10 kg of dried whole fruiting body powder, which was subsequently used for encapsulation. The polysaccharide fruiting body extract contained in the supplement was determined using a β-Glucan Assay Kit (Megazyme, Ireland) and expressed as total glucans (α + β). The selection of β-glucans as marker compounds is justified by their recognized role as quality and activity indicators in mushroom-based products. Authentication of the raw materials, quality control procedures, and batch-to-batch standardization are performed by the manufacturer in accordance with Good Manufacturing Practice (GMP) standards. Voucher specimens are maintained by the manufacturer as part of internal quality documentation. The product was prescribed as a commercially available supplement and was not manufactured or modified for research purposes. Patients were supplemented with 2 capsules per day.

**TABLE 1:** Mycotherapy U-CARE supportive treatment.

Natural compound	Amount (mg) per cap	Dosage
*Agaricus Blazei Murril*	150	2 caps/day
*Cordyceps sinensis*	150	2 caps/day
*Ganoderma lucidum*	150	2 caps/day
*Grifola frondosa*	150	2 caps/day
*Lentinula edodes*	150	2 caps/day

### 2.3 Safety assessment and exclusion criteria

Prior to initiation of integrative supplementation, patients underwent clinical evaluation to assess eligibility and potential safety concerns. As this was a retrospective case series derived from routine clinical practice, supplementation was prescribed only in the absence of known contraindications.

Exclusion criteria for integrative supplementation included:

- Known hypersensitivity or allergy to mushrooms or fungal-derived products;
- Active autoimmune disorders requiring systemic immunosuppressive therapy;
- Severe hepatic impairment;
- Pregnancy or breastfeeding;
- Participation in experimental pharmacological protocols;
- Any clinical condition deemed by the treating oncologist to pose potential risk for supplement use.

Potential drug–drug (DDI) and drug–herb interactions (DHI) were evaluated using the Drug Interactions Checker available on Drugs.com (DRUGS, 2025) and the Medscape Drug Interaction Checker (Medscape, 2025). All concomitant medications were systematically screened for potential interactions with the mycological preparation, including anticancer therapies, drugs prescribed for comorbid conditions, and any additional dietary supplements reported by the patients. Identified interactions were categorized according to the databases’ clinical severity classifications (“contraindicated,” “serious–use alternative,” “monitor closely,” “minor,” or “no interaction found”). A formal database-based interaction assessment was performed in four patients prior to initiation of mycotherapy at the discretion of the treating physician, particularly in cases involving more complex concomitant pharmacological treatments. In the remaining two cases, patients were receiving limited concomitant medications and no clinically relevant interaction concerns were identified during clinical evaluation. Overall, no clinically significant drug–drug or drug–herb interactions involving the U-CARE preparation were identified in this series.

Patients were monitored through routine hematologic, hepatic, and renal laboratory assessments performed as part of standard oncologic care. Attention was given to neutrophil counts, liver enzymes (AST, ALT), bilirubin, and creatinine levels. No adverse events attributable to the mycological preparation were recorded during the observation period. The supplement was administered at the manufacturer’s recommended dosage (2 capsules daily), and no dose escalation was performed. Quality of life observations were based on patient-reported outcomes collected during routine clinical consultations and were not assessed using standardized validated questionnaires.

## 3 Case series

**FIGURE 1:**
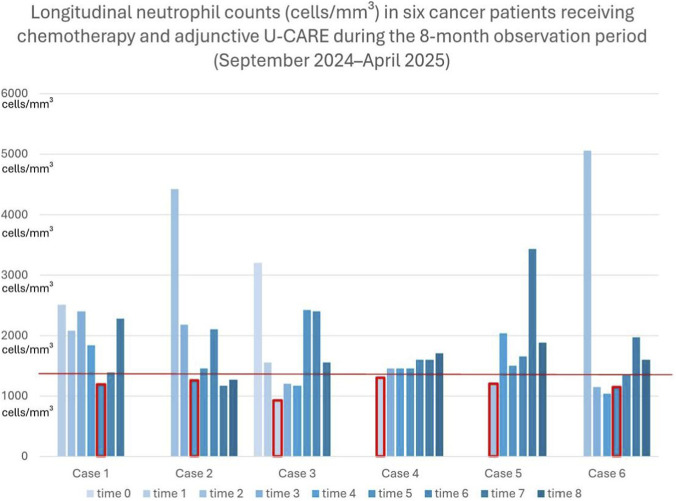
Patients started chemotherapy administration at time 0. Neutrophil counts were assessed every 28 days (Time 0 to 8). The red marker highlights the point at which sustained neutropenia prompted the initiation of Micotherapy U-CARE. The horizontal reference line indicates the threshold used to define neutropenia (1,500 neutrophils/mm^3^).

At the time of publishing 54 patients underwent supportive treatment by an expert team of physicians (Greenlee et al., 2017). From the larger cohort of 54 patients previously reported, six cases were retrospectively selected due to the presence of clinically relevant hematologic toxicity and complete longitudinal laboratory documentation. Four patients had a histologically confirmed diagnosis of ER+ (estrogen receptor-positive), PgR+ (progesteron receptor-positive), breast cancer. Three of them started first-line standard therapy according to international guidelines with a CDK4-6 inhibitor in combination with hormone therapy and one patient was treated oncologically with Trastuzumab Emtansine (TDM1). The fifth patient had pancreatic cancer and underwent standard chemotherapy, while the sixth case was a patient with astrocytoma IDH1-mutated who underwent concomitant chemoradiation therapy (CRT). During treatment, they developed hematologic toxicity ranging from persistent G1 neutropenia to G3 neutropenia and G3 pancytopenia, requiring temporary suspension of oncologic treatment for several cycles. All patients were supported with mycotherapy U-CARE to reduce hematologic toxicity and manage treatments. A detailed overview of the patients’ clinical characteristics can be found in Table 2. Figure 1 presents the longitudinal month-by-month evolution of neutrophil counts for all the cases described. Neutrophil values show fluctuations during treatment; however, they were generally maintained above the neutropenia threshold indicated in figure (1,500 neutrophils/mm^3^), allowing continuation of oncologic therapy. The observation period for neutrophil trends was 8 months, spanning from September 2024 to April 2025, during which neutrophil count were monitored as part of routine oncologic follow-up. Although the trend does not show a progressive increase, it is important to note that the absolute neutrophil count remained above the minimum threshold required to continue treatment.

**TABLE 2:** Demographic and clinical characteristics of included patients. Toxicities were reported according to NCI-CTCAE v5.0.

Variable	Case 1	Case 2	Case 3	Case 4	Case 5	Case 6
Age at diagnosis	55	62	50	62	50	33
Gender	Female	Female	Female	Female	Male	Female
PS (ECOG)	0	1	0	0	1	1
Tumor site	Breast	Breast	Breast	Breast	Pancreas	CNS
Histology	IDC G3	IDC G3	IDC G3	IDC G2	Adenocarcinoma	Astrocytoma IDH1 mutate
Immunophenotype	Luminal A	Luminal B	Luminal B	Luminal B HER2 enriched	-	-
Stage at mycotherapy initiation	III	IV	IV	IV	IV	WHO grade 2
Adjuvant therapy	EC x 4 cycles → PTX x 12 cycles → Abemaciclib + Letrozole for 2 years	​	​	​	​	TMZ + RT → TMZ
First line treatment	-	Palbociclib + Letrozole	Palbociclib + Fulvestrant	TDM-1	Modified FOLFIRINOX	-
Best response to treatment	-	CR	CR	SD	SD	CR
Site of metastasis	-	Skin, lymph nodes	Bone	Lymph nodes, lung	Liver	-
Toxicities (NCI-CTCAE v5.0)	Neutropenia G2	Neutropenia G2 Thrombocytopenia G1	Neutropenia G2	Neutropenia G2	Neutropenia G1	Pancytopenia G3
Chemotherapy cycle at which neutropenia appeared	5 months (5th cycle)	3 months (3rd cycle)	3 months (3rd cycle)	NA	1 month (2nd cycle	4 months (4th cycle)
Dose reduction	100 mg abemaciclib	100 mg palbociclib	100 mg palbociclib	-	-	-
Treatment duration	10 months, ongoing at data cut-off	14 months, ongoing at data cut-off	4 years	4 years, ongoing at data cut-off	1 year, ongoing at data cut-off	13 months, ongoing at data cut-off
U-care exposure	8 months	11 months	4 years	2 years	11 months	9 months
DDI and DHI	Yes	Yes	Yes	-	Yes	-

CR, complete response; CTCAE, Criteria for Adverse Events following Common Terminology Criteria for Adverse Events Version 5.0; CNS, central nervous system; DDI, drug-drug interaction; DHI, drug-herbs interaction; EC → PTX: Epirubicine + Ciclophosfamide → Paclitaxel; IDC, infiltrating ductal carcinoma; SD, stable disease; TMZ + RT → TMZ (STUPP regimen): concomitant chemio-radiotherapy with Temodal followed by Temodal in monotherapy.

### 3.1 Case 1

A 55-year-old female patient underwent central quadrantectomy and axillary dissection in March 2024 for invasive ductal carcinoma of the breast (pT3pN1, Luminal A). Following surgery, she received adjuvant chemotherapy according to the EC (Epirubicin and Cyclophosphamide) regimen for four cycles, followed by weekly paclitaxel for 12 weeks, and subsequent complementary radiotherapy. The chemotherapy was well tolerated, with no significant toxicities. Although hematologic parameters remained at the lower limit of the normal range, no treatment interruptions were required. The final chemotherapy cycle was completed on 31 October 2024. Given the high risk of recurrence, adjuvant endocrine therapy with letrozole and the CDK4/6 inhibitor abemaciclib (150 mg twice daily) was initiated on 14 November 2024. During the second cycle of abemaciclib, the patient developed grade 2 neutropenia, prompting a dose reduction to 100 mg twice daily on 20 January 2025. Considering the trend in her white blood cell counts, adjunctive mycotherapy with U-CARE was proposed. Before initiation, a DDI-DHI interaction test was performed to exclude potential drug–supplement interactions (Mao et al., 2022; Zhang et al., 2023). The test revealed no contraindications for the concurrent use of U-CARE with abemaciclib or proton pump inhibitors (PPIs) that the patient was already taking. Following the introduction of U-CARE mycotherapy, the patient was able to continue abemaciclib therapy without further interruptions, maintaining good tolerability and quality of life. Over a 10-month treatment period, the neutrophil count progressively remained above the minimum threshold required for treatment continuation (WBC: 2,500/mm^3^, N: 59%).

### 3.2 Case 2

A 75-year-old female patient underwent surgery in 2011 for multicentric invasive ductal carcinoma of the breast (G3, pT1cN1mi). During the same period, she also underwent a right equatorial quadrantectomy for ductal carcinoma *in situ* (pTis). Postoperatively, she received adjuvant endocrine therapy with letrozole for 5 years. In December 2021, following the diagnosis of skin metastases confirmed by biopsy, first-line therapy with palbociclib in combination with letrozole was initiated and is still ongoing. The treatment has been well tolerated, with adverse events consistent with those reported in pivotal clinical trials. Specifically, the patient developed persistent grade 2 neutropenia and grade 1 thrombocytopenia during palbociclib therapy. Typically, such hematologic toxicity necessitates temporary interruption of treatment until recovery. To support hematologic function and improve treatment adherence, mycotherapy with U-CARE was introduced. Prior to initiation, a DDI-DHI interaction test was performed to exclude potential drug–supplement interactions. The test revealed no contraindications for the concomitant use of U-CARE with palbociclib or the beta-blockers the patient was taking. The U-CARE mycotherapy was well tolerated and associated with improved absolut neutrophil count and overall quality of life. Quality of life observations were based on patient-reported outcomes collected during routine clinical consultations and were not assessed using standardized validated questionnaires. Importantly, the most recent FDG-PET/CT scan performed in September 2025 demonstrated a complete metabolic response, defined as the absence of metabolically active disease according to PERCIST imaging criteria.

### 3.3 Case 3

A 57-year-old female patient was diagnosed in February 2017 with invasive ductal carcinoma of the left breast (G3, Luminal B) following a core needle biopsy. She received neoadjuvant chemotherapy with the AC regimen (Adriamycin and Cyclophosphamide) for four cycles, achieving a partial response. Subsequently, she underwent a left mastectomy, followed by adjuvant chemotherapy with weekly docetaxel for 12 weeks and radiotherapy to the chest wall. In November 2017, adjuvant endocrine therapy with letrozole was initiated. In October 2018, the patient was diagnosed with bone metastasis in the thoracic spine (dorsal region), confirmed by bone biopsy. She received local radiotherapy to the affected area and started first-line systemic therapy with palbociclib in combination with fulvestrant, which she continues to date. From February 2019 to July 2020, the patient received palbociclib at a dose of 125 mg daily. Due to persistent grade 2 neutropenia, the dose was reduced to 100 mg daily from July 2020 until March 2025. Throughout treatment, white blood cell and neutrophil counts remained at the lower limit of the normal range, and therapy was temporarily interrupted four times for 1 week each because of hematologic toxicity. To prevent further treatment interruptions, a DDI-DHI interaction test was conducted before introducing supportive mycotherapy with U-CARE. The patient was not taking any additional medications, and the test confirmed no harmful interactions between U-CARE and her oncologic treatment. Following the initiation of U-CARE mycotherapy, the patient’s white blood cell counts improved, allowing continuous administration of the CDK4/6 inhibitor and fulvestrant without further interruptions. The patient also reported reduced fatigue and improved QoL.

### 3.4 Case 4

A 62-year-old female patient with a history of invasive ductal carcinoma of the breast, luminal B HER2 enriched, was initially diagnosed and surgically treated in 1997. In 2005, lung metastases were detected and managed with stereotactic radiotherapy. Following disease progression in 2017, the patient received first-line systemic therapy with pertuzumab and trastuzumab, which she continued for approximately 2 years. In 2018, second-line therapy with trastuzumab emtansine (T-DM1) was initiated. During the COVID-19 pandemic in 2021, the patient received two doses of the Pfizer–BioNTech mRNA vaccine and subsequently developed persistent grade 2 neutropenia. Given the hematologic toxicity and the risk of having to interrupt the ongoing oncologic treatment, supportive mycotherapy with U-CARE was introduced to stabilize blood counts and maintain treatment continuity. Remarkably, following the initiation of U-CARE, the patient’s neutrophil levels remained above the minimum threshold required for treatment continuation, allowing her to continue T-DM1 therapy without interruption. No further hematologic toxicities were reported, and the patient experienced an overall improvement in clinical condition, energy levels, and QoL. In October 2022, due to suboptimal tolerance to T-DM1 and radiologic disease stability, the patient was transitioned to trastuzumab plus exemestane. Despite this, lung disease progression was documented in March 2023 and confirmed histologically (ER 100%, PgR 2%, Ki-67 14%, HER2 score 0). She subsequently entered an Early Access Program (EAP) and began treatment with trastuzumab deruxtecan (T-DXd). Further disease progression prompted sequential therapies, including a CDK4/6 inhibitor, pegylated liposomal doxorubicin (November 2024), sacituzumab govitecan (April 2025), and finally another cycle of pegylated liposomal doxorubicin (30 mg/m^2^ every 21 days), administered with or without oral cyclophosphamide in September 2025.

### 3.5 Case 5

A 52-year-old male presented with jaundice and was admitted for further evaluation. Abdominal ultrasound and CT imaging revealed a pancreatic mass. On 29 February 2024, he underwent a pylorus-preserving, robot-assisted pancreaticoduodenectomy. Histopathological examination (19 March 2024) confirmed a moderately differentiated pancreatic ductal adenocarcinoma, with metastatic involvement of 9 out of 31 lymph nodes. The postoperative course was complicated by a catheter-related bloodstream infection requiring targeted antibiotic therapy, delayed gastric emptying with partial ulceration at the duodenojejunal anastomosis, and the need for parenteral nutrition. On 2 May 2024, tumor markers were markedly elevated (CA 19.9: 6,061 U/mL; CEA: 43.28 ng/mL). A CT scan on 8 May 2024, demonstrated multiple hypodense liver lesions up to 4.2 cm, consistent with metastatic disease. Given the advanced stage and clinical condition, first-line chemotherapy with FOLFIRINOX (Oxaliplatin 85 mg/m^2^, Irinotecan 150 mg/m^2^, Folinic Acid 400 mg/m^2^, and 5-FU 2,400 mg/m^2^ continuous infusion over 46 h, every 14 days) was initiated. After the ninth cycle, the patient developed persistent grade 2 neutropenia, resulting in treatment delay. To promote neutrophil recovery and prevent further interruptions, mycotherapy with U-CARE was introduced following a DDI/DHI interaction test, which confirmed no harmful interactions with chemotherapy or concomitant PPI therapy. The addition of U-CARE led to stabilization of hematologic parameters, enabling the continuation of chemotherapy without further delays until February 2025, when treatment was switched to FOLFIRI, maintaining concomitant U-CARE support. At the last follow-up in September 2025, CT imaging demonstrated stable disease (SD) and both CEA and CA19.9 tumor markers were negative. The patient reported a notable improvement in clinical condition, energy levels, and overall QoL throughout the course of treatment with ongoing mycotherapy support. Notably, this case illustrates how an integrative oncology approach may play an indirect yet meaningful role in reducing chemotherapy-related toxicity, thereby improving treatment adherence and sustaining therapeutic intensity. Such supportive interventions can ultimately enhance clinical outcomes and health-related QoL, reinforcing the value of evidence-based complementary care within conventional oncologic management.

### 3.6 Case 6

A 33-year-old female patient presented in August 2023 with seizures and loss of consciousness. Brain MRI revealed a left frontotemporal–insular mass. In March 2024, she underwent microsurgical resection of the lesion, and histopathological examination confirmed an IDH1-mutated grade 2 astrocytoma. In May 2024, the patient initiated concomitant chemoradiotherapy with temozolomide, followed by temozolomide monotherapy starting in August 2024. During treatment, she was hospitalized three times for grade 3 pancytopenia, requiring blood transfusions and supportive care. On two of these admissions, she developed septic shock secondary to *Klebsiella pneumoniae* infection, which was successfully managed with targeted antibiotic therapy. After discharge, supportive mycotherapy with U-CARE was introduced to improve hematologic recovery and treatment tolerance. Following the introduction of U-CARE, the patient experienced a progressive remained above the minimum threshold required for treatment continuation of hematologic parameters, resolution of pancytopenia, and a marked improvement in overall clinical condition and QoL. She was able to continue temozolomide therapy without further interruptions. At disease reassessment in February 2025, MRI imaging showed no radiologic or clinical signs of relapse, confirming stable disease under continued temozolomide treatment in combination with U-CARE mycotherapy. In the months following this evaluation, the patient relocated to another region, and no further follow-up data are available.

## 4 Discussion

This case series reports six notable cases from a previously published study involving cancer patients receiving supportive care from a multidisciplinary team (Berretta et al., 2025). In these patients, the integration of complementary and integrative medicine (CIM), including the mycotherapy blend U-CARE, was associated with improved health-related quality of life (QoL) and better management of treatment-related adverse events, particularly hematologic toxicity such as neutropenia. Several patients experienced grade 3 and/or persistent grade 1–2 neutropenia leading to dose reductions or treatment delays. Following supplementation with U-CARE, anticancer therapy could be continued without further modifications or interruptions, with concurrent recovery of blood counts. Although causal relationships cannot be established, these observations suggest that conventional oncology and CIM approaches may coexist synergistically when supervised by trained healthcare professionals.

Among CIM modalities, medicinal mushrooms have gained increasing attention as a potential resource in oncology due to their immunomodulatory and oncoimmunologic properties (Ikram et al., 2025). Their biological activity is attributed to several bioactive compounds, including polysaccharides (e.g., β-glucans), polysaccharide–protein complexes, triterpenes, steroids, polyphenols, and alkaloids, which can modulate immune responses and interfere with dysregulated signaling pathways involved in tumor proliferation, survival, invasion, and angiogenesis (Bentharavithana et al., 2025; Quagliariello et al., 2017). Certain compounds, such as cordycepin and hispolon, have demonstrated anticancer activity and the ability to enhance the effects of conventional therapies, particularly in drug-resistant contexts (Khan and Tania, 2020). In addition, medicinal mushrooms have been reported to support immune function by promoting lymphocyte proliferation and differentiation. For example, increases in CD8^+^ T cells and CD19^+^ B cells have been observed in breast cancer patients receiving mycotherapy (Gariboldi et al., 2023), while Reishi supplementation in lung cancer patients has been associated with increased levels of cytokines such as IFN-γ and IL-2, as well as enhanced natural killer cell activity (Zhao et al., 2020).

Another emerging area of interest is the role of the gut microbiota in cancer progression, treatment response, and toxicity. Dysbiosis has been associated with inflammation, immune dysregulation, and altered metabolic pathways that may influence both chemotherapy tolerance and therapeutic outcomes (Scano et al., 2025). Conversely, interventions targeting the microbiota, including dietary strategies and complementary and integrative medicine approaches, may help support immune homeostasis and potentially improve responses to anticancer therapies (Incognito et al., 2025). However, commonly prescribed medications such as antibiotics and proton pump inhibitors can significantly alter the intestinal microbiota, potentially affecting immune responses and treatment efficacy. Awareness of these interactions is therefore important when managing patients receiving integrative interventions (Ciapp et al., 2025).

The clinical relevance of CIM in oncology is increasingly recognized by international guidelines. In 2018, the American Society of Clinical Oncology (ASCO) published recommendations for integrative therapies in breast cancer patients, providing evidence-based guidance for their safe and appropriate use (Lyman et al., 2018). Similarly, European health policies have acknowledged the importance of integrative and patient-centered approaches to cancer care, emphasizing the potential contribution of scientifically validated complementary practices when delivered under professional supervision (TRILLET-LENOIR V, 2022).

Beyond medicinal mushrooms, CIM may include herbs, vitamins, minerals, and probiotics. For example, vitamin C supplementation has been associated with improvements in quality of life in chronic diseases (Berretta et al., 2020), while vitamin D status has been linked to improved progression-free survival in breast cancer patients undergoing neoadjuvant therapy (Ottaiano et al., 2024). However, the increasing use of CIM among cancer patients underscores the need for careful clinical oversight. Physicians should ensure adherence to evidence-based oncologic treatments, evaluate potential drug–supplement interactions, and encourage patients to disclose all complementary products they use (Berretta et al., 2022). Although no adverse events attributable to the investigated supplement were observed in this case series, the safety of medicinal mushroom preparations should be interpreted cautiously, as potential risks may include allergic reactions, gastrointestinal discomfort, or immunomodulatory effects in susceptible individuals.

Several limitations must be acknowledged. This report is based on a small case series without a comparator arm, preventing causal inference and limiting generalizability. The findings should therefore be interpreted as observational and hypothesis-generating, reflecting associations observed in routine clinical practice rather than definitive evidence of efficacy. Future research should include well-designed randomized controlled trials, validated quality-of-life assessments, and prospective biomarker analyses to better clarify the efficacy, safety, and mechanisms of integrative mycotherapy in oncology.

This study was conducted with consideration of the Four Pillars of Best Practice in Ethnopharmacology. The pharmacological rationale for medicinal mushrooms is supported by preclinical and clinical evidence describing the immunomodulatory and hematopoietic effects of β-glucans and related compounds (Ikram et al., 2025; Bentharavithana et al., 2025; Quagliariello et al., 2017; Khan and Tania, 2020; Gariboldi et al., 2023; Zhao et al., 2020). The investigated preparation is a commercially registered dietary supplement, with fungal species taxonomically validated according to Index Fungorum and produced according to declared manufacturing standards. From a clinical perspective, this report presents real-world observations focusing on outcomes relevant to oncologic practice, such as treatment continuity, neutrophil recovery, and patient-reported QoL. Finally, the preparation consists of cultivated fungal species and does not involve endangered taxa according to manufacturer declarations.

## 5 Conclusion

The six clinical cases presented here exemplify an integrative approach based on shared decision-making between patients and a multidisciplinary team. Supportive therapy with medicinal mushrooms was selected according to current clinical evidence and established guidelines (Lyman et al., 2018) allowing patients to continue oncologic treatment without experiencing additional complications or dose interruptions. This approach highlights the potential of combining conventional oncology with complementary interventions in a structured and closely supervised setting. Importantly, integrative strategies such as mycotherapy may exert an indirect yet clinically meaningful role by reducing treatment-related toxicity, thereby improving patients’ tolerance to therapy. This reduction in adverse events can in turn enhance adherence to oncologic regimens, sustain treatment intensity, and ultimately contribute to improved therapeutic efficacy and QoL. While these preliminary findings are encouraging, it is important to acknowledge the limitations inherent to a small case series. The sample size restricts the generalizability of the results and precludes definitive conclusions regarding efficacy and safety. Larger, well-designed prospective clinical trials will therefore be necessary to validate these observations and to establish standardized protocols for integrating mycotherapy into oncology care. Nonetheless, the present cases suggest that mycotherapy may serve as a promising supportive option, with multiple potential benefits. These include mitigating common treatment-related adverse events such as neutropenia, supporting immune function, improving patient adherence to anticancer regimens, and enhancing overall health-related quality of life. Additionally, given the immunomodulatory properties of medicinal mushrooms, there is a potential for synergistic effects with conventional therapies, which may ultimately contribute to improved oncologic outcomes. Future research should also explore the interplay between mycotherapy, the gut microbiota, and host immunity, as this may further refine patient-tailored supportive strategies. In conclusion, these cases reinforce the feasibility and potential value of integrative oncology approaches that are evidence-informed, patient-centered, and multidisciplinary, emphasizing the importance of continued investigation into complementary therapies as adjuncts to standard cancer care.

## Data Availability

The original contributions presented in the study are included in the article/supplementary material, further inquiries can be directed to the corresponding author.
